# Identifying genetic variants associated with amphotericin B (AMB) resistance in *Aspergillus fumigatus* via *k-*mer*-*based GWAS

**DOI:** 10.3389/fgene.2023.1133593

**Published:** 2023-05-09

**Authors:** Meng-Meng Chen, Guo-Hui Shi, Yi Dai, Wen-Xia Fang, Qi Wu

**Affiliations:** ^1^ State Key Laboratory of Mycology, Institute of Microbiology, Chinese Academy of Sciences, Beijing, China; ^2^ University of Chinese Academy of Sciences, Beijing, China; ^3^ Guangxi Biological Sciences and Biotechnology Center, Guangxi Academy of Sciences, Nanning, Guangxi, China

**Keywords:** *Aspergillus fumigatus*, AMB, resistance, k-mers, GWAS

## Abstract

*Aspergillus fumigatus* is one of the most common pathogenic fungi, which results in high morbidity and mortality in immunocompromised patients. Amphotericin B (AMB) is used as the core drug for the treatment of triazole-resistant *A. fumigatus*. Following the usage of amphotericin B drugs, the number of amphotericin B-resistant *A. fumigatus* isolates showed an increasing trend over the years, but the mechanism and mutations associated with amphotericin B sensitivity are not fully understood. In this study, we performed a *k*-mer-based genome-wide association study (GWAS) in 98 *A. fumigatus* isolates from public databases. Associations identified with *k*-mers not only recapitulate those with SNPs but also discover new associations with insertion/deletion (indel). Compared to SNP sites, the indel showed a stronger association with amphotericin B resistance, and a significant correlated indel is present in the exon region of *AFUA_7G05160*, encoding a fumarylacetoacetate hydrolase (FAH) family protein. Enrichment analysis revealed sphingolipid synthesis and transmembrane transport may be related to the resistance of *A. fumigatus* to amphotericin B. The expansion of variant types detected by the *k*-mer method increases opportunities to identify and exploit complex genetic variants that drive amphotericin B resistance, and these candidate variants help accelerate the selection of prospective gene markers for amphotericin B resistance screening in *A. fumigatus*.

## Introduction

*Aspergillus fumigatus* is one of the important pathogenic fungi, accounting for more than 60% of clinical isolates (Steinbach et al., 2012). The sporulation ability of *A. fumigatus* is very strong, producing thousands of conidia per spore head. These spores are very small, 2–3 μm in diameter, and are easily inhaled into the alveoli by humans (Latgé, 1999). Each person inhales several hundred conidia per day (Hospenthal et al., 1998). In normal people, spores inhaled into the body can be promptly eliminated by the body’s own immune system. However, in immunodeficient patients, the inhaled spores are not completely cleared. The germinated mycelium can invade blood vessels and then spread throughout the body with the blood. According to statistics, more than 200,000 cases of invasive aspergillosis occur globally each year (Brown et al., 2012), and the mortality rate of invasive aspergillosis ranges from 60% to 90% (Tekaia and Latge, 2005).

The first-line drugs for the treatment of invasive aspergillosis are triazole drugs, but resistance of *A. fumigatus* to triazoles is currently a widespread problem. Amphotericin B (AMB) is recommended as a core therapy for invasive aspergillosis that develops triazole resistance (Verweij et al., 2015). AMB is a class of polyene antifungal drugs that act by irreversibly binding to ergosterol on cell membranes, leading to damage to membrane permeability and leakage of important intracellular substances, eventually resulting in fungal cell death (Gray et al., 2012). Some studies have also suggested that the role of AMB is linked to oxidative killing mechanisms (Anderson et al., 2014). AMB induced cell membranes’ oxidative damage and DNA damage through the production of reactive oxygen species (ROS), leading to decreased cell viability. With the wide application of AMB, AMB-resistant isolates of *A. fumigatus* gradually appeared. A recent review found that 353 (2.01%) of 17,494 *A. fumigatus* isolates were AMB-resistant, and the number of AMB-resistant isolates showed an increasing trend from 2016 to 2020 (Fakhim et al., 2022). Previous studies have suggested that fungal resistance to AMB may be related to the glucan content in the cell wall or ergosterol content in cell membranes (Walsh et al., 2003; Waness et al., 2009). In addition, Geraghty and Kavanagh (2003) discovered AMB resistance may be associated with decreased mitochondrial activity and reactive oxygen intermediate (ROI) production. However, the mechanism of AMB resistance in *A. fumigatus* is currently unknown, and resistance-associated variant loci are largely unexplored.

A genome-wide association study (GWAS) can be used to identify candidate genomic variant loci associated with phenotypes. The commonly used genetic markers are single nucleotide polymorphisms (SNPs), and SNP-based GWAS has been widely used to detect genetic variations associated with fungal drug resistance (Fan et al., 2021). However, numerous studies have found many structural variants (SVs), which often lead to phenotypic variations. In recent years, an alignment-free method has been developed by using the presence/absence pattern sequences of constant length *k* (*k*-mer) that are directly associated with phenotypes. Associations identified with *k*-mers not only recapitulate those found with SNPs but also discover new associations with structural variants and deletion regions of the reference genomes (Rahman et al., 2018; Voichek and Weigel, 2020). In this study, we obtained raw sequencing data from public databases for *A. fumigatus* from nine countries, generated *k-*mers from the assembled genome sequences, and correlated with the presence/absence status of *k-*mers in the strains with the minimum inhibitory concentration (MIC) of AMB against the strains, with the aim of finding the variants that lead to resistance to AMB in *A. fumigatus* and to analyze the associated resistance mechanisms.

## Materials and methods

### Source of strains

This study used 98 published strains of *A. fumigatus* with known resistance to AMB. They came from nine countries, 82 from the clinical environment and 16 from the natural environment. The raw reads’ sequences were downloaded from the NCBI SRA (https://www.ncbi.nlm.nih.gov/sra) database. The SRA numbers and relevant information such as the minimum inhibitory concentration (MIC) of AMB are given in Supplementary Table S1 in the Appendix.

### Genome assembly

Raw reads were trimmed to remove low-quality (Q < 20), ambiguous, and adaptor bases using fastp (Chen et al., 2018). Then, the high-quality reads were compared to the reference genome (GCF_000002655.1) using BWA-MEM (Li, 2013). The genome of strains was *de novo* assembled using SOAPdenovo-127mer (Luo et al., 2012). The configuration file is set separately for each strain, with “max_rd_len” and “rd_len_cutoff” set to the average read length of the strain; “avg_in” is set to insert fragment length, “reverse_seq = 0,” “asm_flags = 3,” “rank = 1,” “pair_num_cutoff = 3,” and “map_len = 35.” The parameter “-K” representing the *k*-mer length and its value is in the range of 55%–85% of the average length of the strain’s reads in this study. The quality of the genome assembly was assessed using QUAST (Gurevich et al., 2013), and a higher N50 was selected for further analysis. After assembling, the median N50 was 50,430 bp and the median genome size was about 28 Mb for all strains.

### Counting *k-*mers in the *de novo* assembled genome

**FIGURE 1:**
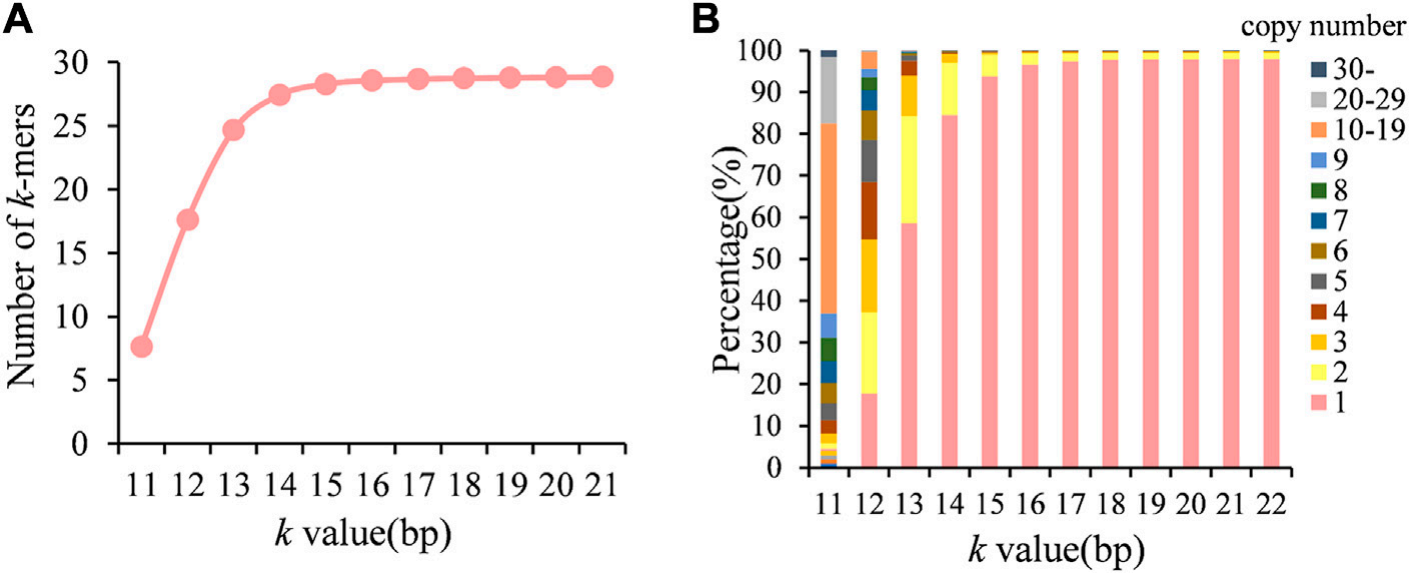
Number of *k-*mer type and distribution of copy numbers in the genome at different *k-*values. **(A)** Number of *k-*mer types; **(B)** distribution of *k*-mer copy numbers.

In order to select the appropriate *k-*mer length, we first counted the number of *k-*mers in the genome of *A. fumigatus* at different *k-*values. As shown in Figure 1A, the *k-*mer type gradually increased as the *k-*value increased but reached a plateau when k > 17. As the *k-*value increased, the *k-*mers copy number was decreased, and when *k* = 19, about 98% *k-*mers was unique in the genome (Figure 1B). Therefore, the optimal *k-*value in this study was 19.

Having identified the optical *k-*value, we extracted the *k-*mers from each genome and then used our own developed *k-*merToMarix program to combine the set of *k-*mers of all strains into a *n* × *m* matrix, where *n* was the number of strains and *m* was the number of *k-*mers. The interior of the matrix is the occurrence number of *k-*mers in the strain. The *k*-mers with multiple copies in the genome are generally derived from duplications and the possible presence of multiple copies of mobile elements within the genome and, thus, was filtered out to avoid assemble errors. After filtering, the majority of *k*-mers possess two states: present once or absent.

### Reference-based alignment and variant calling

The clean paired reads were mapped to the reference genome using BWA with default parameters. SAMtools was used to convert the alignment results into the BAM files, and Picard-tools (http://picard.sourceforge.net) was used to remove the duplicated sequence (Li et al., 2009). SNPs were detected using the mutation analysis software GATK and further filtered based on quality value, depth, repeatability, and other factors (QD < 2.0, ReadPosRankSum < −8.0, FS > 60.0, OUAL<30.0, DP < 4.0, MQ < 40.0, and MappingQualityRankSum < −12.5) (McKenna et al., 2010). The final 487,348 high-confidence variant loci were obtained. The variant loci were annotated using ANNOVAR based on the genome annotation file for *Af*293 (assembly ASM265v1) (Wang et al., 2010).

### Genome-wide association study (GWAS)

GWAS based on *k-*mers and SNPs were performed using Gemma (Zoubarev et al., 2012), and the MIC of AMB against strains was used as the phenotypic value to calculate a standardized association matrix; the Wald test was used. Quality control of genetic markers is required when performing the GWAS, and usually, only markers with a minor allele frequency (MAF) greater than 0.05 or 0.1 are retained as those with very low MAF are susceptible to bias in the genotype calling algorithm. The SNP sites with an MAF less than 0.1 and with a maximum of 5% missing values (-miss 0.05) were removed, which finally leaves 149,962 SNP loci for the GWAS. Using presence and absence of a *k*-mer as two allelic contrasts, the *k*-mers with minor allele frequencies less than 0.1 were first filtered out. The *k-*mer matrix was then converted to VCF format using our own developed *k-*merToVcf program, and the VCF format files were converted to binary files ending in bed, .bim, and .fam for the GWAS using PLINK (Purcell et al., 2007). Gemma was then run using the same parameters for the *k*-mer and SNP genome-wide association. In order to find as many variant loci as possible that are associated with AMB resistance, we calculated the polygenic risk score (PRS) in PRSice-2 and found the most appropriate *p*-value as a screening threshold (Choi and O'Reilly, 2019).

### Analysis of *k-*mers’ locations and host genes

For the significant *k-*mers, we first assembled overlapping *k*-mers into fragments and mapped them to corresponding genomes, and then intercepted the 100-bp sequence around it. After mapping their sequence to the reference gene, the exact position of the fragment on the reference genome was obtained. The fragment was aligned to the sequence at the corresponding position on the reference genome to identify the specific variant type. If the fragment could match the reference genome exactly, it was considered that the fragment had SNP-type variation. If there was a gap between the alignment results, it was considered that there was an indel. In addition, we also annotated the fragment against the annotation file of the reference genome to identify associated genes and used the enrichment analysis website DAVID (https://david.ncifcrf.gov/) to perform Gene Ontology (GO) and Kyoto Encyclopedia of Gene and Genome (KEGG) pathway enrichment analyses.

## Results

### The *k-*mer matrix

**FIGURE 2:**
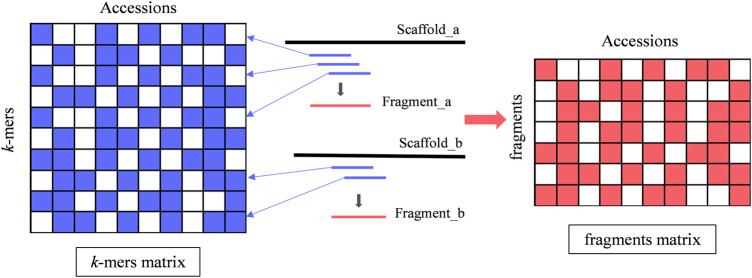
Process of assembling a fragment by *k-*mers. The short blue horizontal line represents the *k-*mer, the blue arrow points to the position in the *k-*mer matrix, and the short red horizontal line represents a fragment assembled from complete linkage *k-*mers. In both the *k-*mer matrix and the fragment matrix, the color-filled cells represent the presence state of a *k-*mer or fragment, and the blank cells represent the absence state.

The number of *k-*mers extracted from 98 strains ranged from 26 million to 32 million. *k-*mers from all strains were combined into 98 × 78,856,507 matrices. After high-frequency filtering, 66,248,564 unique *k-*mers were retained. A schematic representation of the *k-*mers matrix is shown in Figure 2, where accessions represent the 98 strain numbers of *A. fumigatus* and *k-*mers represent the 66,248,564 *k-*mers. *k-*mer is only present or absent in the matrix in two states. After the removal of *k*-mers with MAF <0.1, we finally obtained 6,822,029 *k-*mers for the GWAS. To define a set of *k*-mers most likely to be associated with AMB resistance, we had to set a *p*-value threshold. Unfortunately, a single genetic variant is typically tagged by several *k*-mers, and the Bonferroni threshold would not accurately reflect the effective number of independent tests. To account for non-independence, we introduced an approach to assemble the *k*-mers that were derived from the same variant into a longer fragment (Figure 2). This approach was based on the linkage between adjacent *k*-mers. If *r* ^*2*^ between adjacent *k*-mers was 1, the *k*-mer was derived from the same variants. After assembly, the number of *k*-mer fragments was about tenth of *k*-mers.

### Genome-wide association study

**FIGURE 3:**
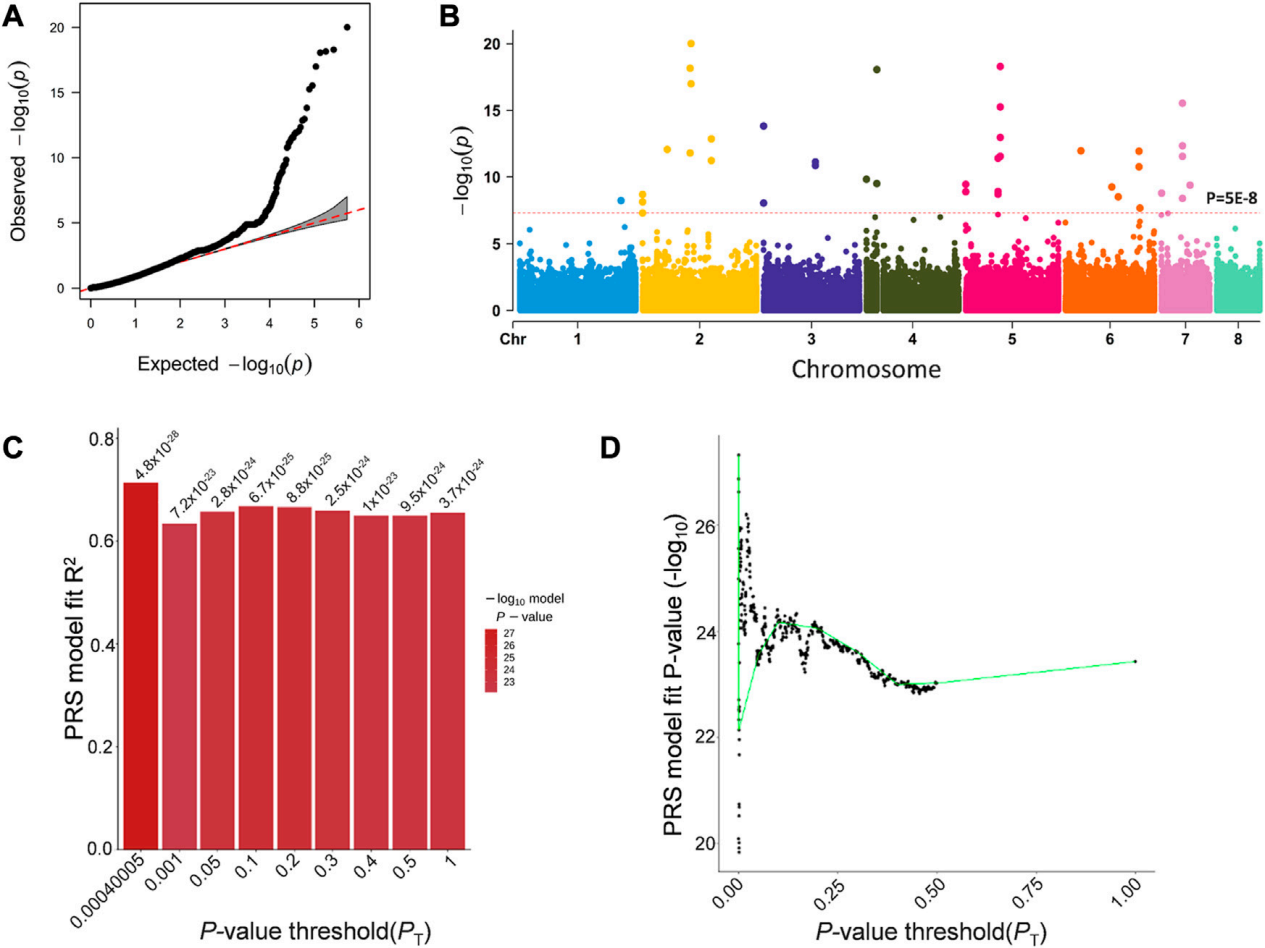
GWAS results for *k*-mers associated with AMB resistance in *A. fumigatus*. **(A)** Quantile–quantile plots of the results of the GWAS. **(B)** Manhattan plot based on the GWAS results for *k-*mers. **(C)** Bar chart for PRSice-2 shows the percentage distribution of the explained PRS risk scores corresponding to the association results obtained for different thresholds, with the highest point of the bar chart indicating that the model is optimal. **(D)** Distribution of *p*-values corresponding to the association results obtained for different thresholds.

In order to find mutations associated with AMB resistance, we performed a *k-*mer-based GWAS analysis of 98 *A. fumigatus* strains, using a MIC of AMB against the strains as the phenotypic value (Figures 3A, B). Based on the results of the GWAS analysis, we calculated the PRS using PRSice-2. The *p*-value with 0.00040005 was under the best predictive model, and 688 *k-*mers with a *p*-value less than this threshold in the GWAS results were defined to be significantly associated with AMB resistance (Figures 3C, D).

**FIGURE 4:**
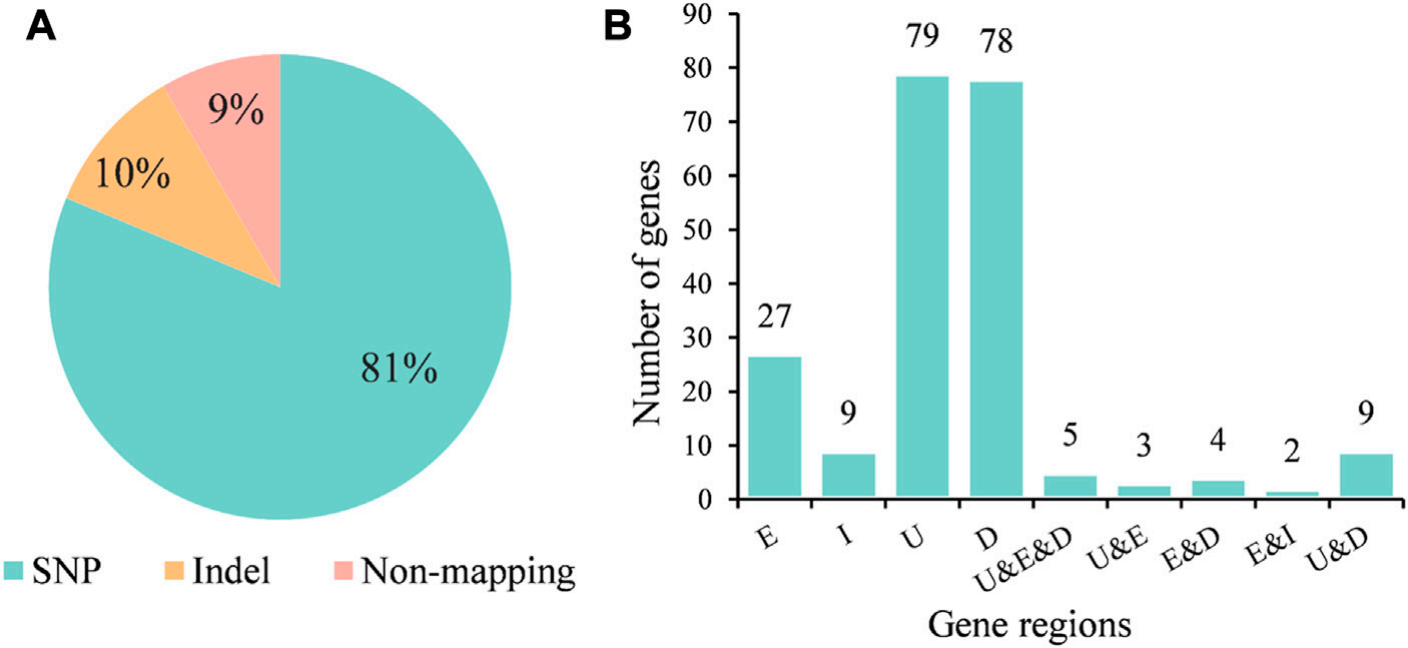
AMB resistance-associated variant types and their regions in genes. **(A)** Proportion of different AMB resistance-associated variant types. **(B)** Number of genes with AMB resistance-associated variants in different regions of genes. E for exon; I for intron; U for upstream; D for downstream.

We analyzed the types of variation that occurred on the 688 *k-*mers fragments and observed that 81% fragments were tagged SNP sites, while 10% of the fragments were derived from indel (Figure 4A). Indel sites showed stronger associations than SNP sites, with nine of the top 10 most significant *k-*mer fragments identifying indel sites and only one identifying SNP sites. We further analyzed their positions on the genes and found the *k*-mers in the exonic regions of 27 genes, in the intron region of 9 genes, in the upstream regions of 79 genes, and in the downstream regions of 78 genes. In addition, we found 104 genes possessed multiple variants, and the upstream, exonic, and downstream regions of five genes (AFUA_1G16410, AFUA_4G02650, AFUA_4G02680, AFUA_4G02700, and AFUA_5G02010) have mutations associated with AMB resistance (Figure 4B). The gene with the highest significance (*p* = 4.05e-10) for mutations on exons was AFUA_7G05160, a fumarylacetoacetate hydrolase family protein, followed by AFUA_1G16410 (*p* = 5.71e-7), a C6 transcription factor. The detailed information about associated genes is given in Supplementary Table S2.

**FIGURE 5:**
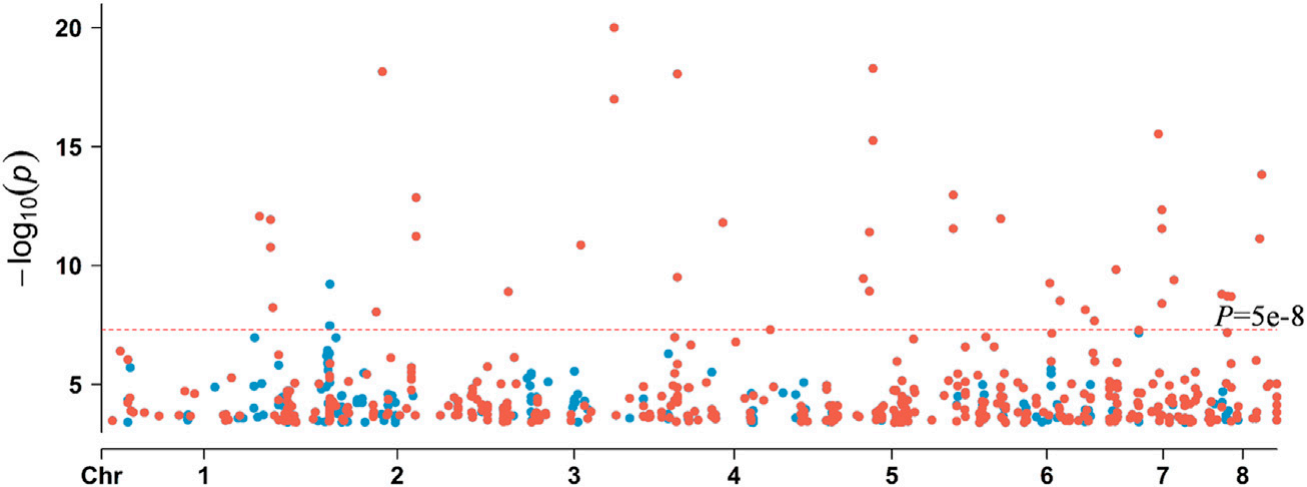
Comparison of the significance of sites in *k-*mer-based and SNP-based GWAS with *p* < 0.00040005. Blue dots represent SNPs, and red dots represent *k-*mers.

**FIGURE 6:**
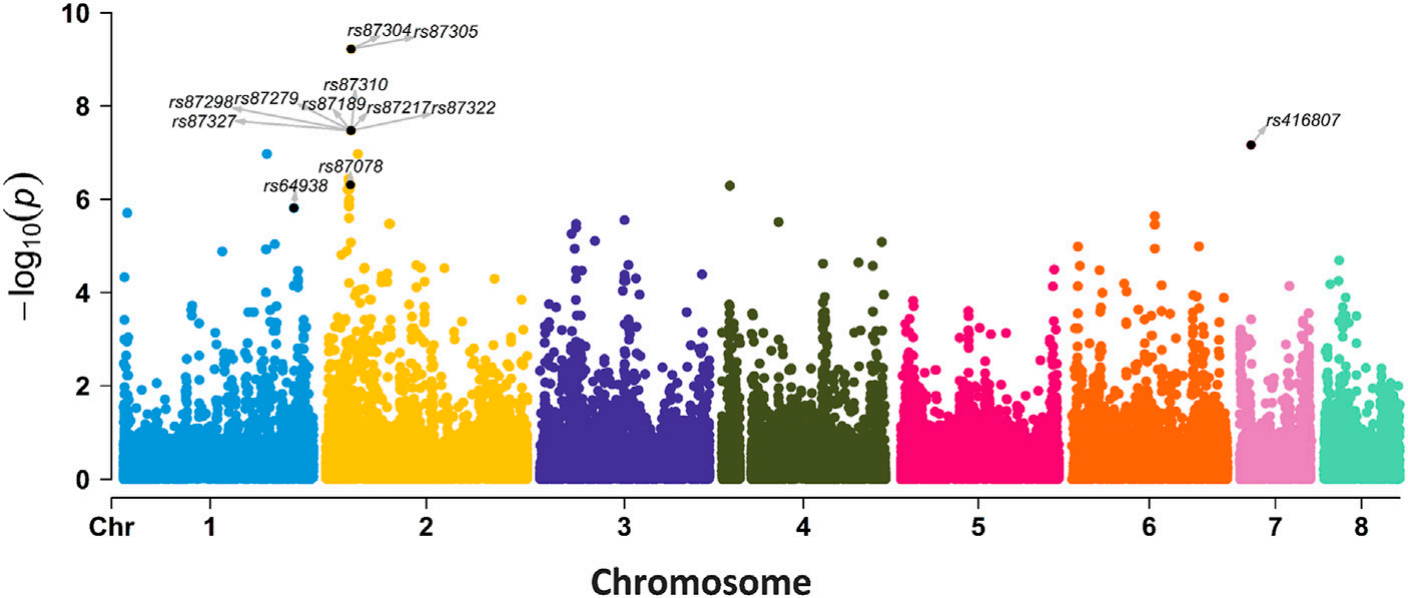
GWAS results for SNPs associated with AMB resistance in 98 *A. fumigatus* strains. The labeled 12 SNPs were also found in *k-*mer-based GWAS with *p* < 0.00040005.

We also performed an SNP-based GWAS using the same parameters as the *k*-mer-based GWAS. The *p*-value of SNP was lower than *k-*mer (Figures 5, 6). The top 12 SNP sites that were identified in the SNP-based GWAS were also identified with *k*-mers, but the *k*-mer showed high statistical support. Further analysis of the 12 SNP sites showed that two SNP sites were located in the exon region of genes, while others were located in the upstream, downstream, or intergenic regions of the genes (Supplementary Table S3). For SNP-based GWAS analysis, 61 SNP sites were significantly associated with AMB resistance (*P* = 1e-5). Of them, 13 SNP sites were non-synonymous mutations. The most significant non-synonymous mutant site was found in AFUA_2G02360, which encodes UDP-glucose: glycoprotein glucosyltransferase, and the deletion of this gene is associated with alterations in the fungal cell wall. Only AFUA_2G02210 contained two non-synonymous mutant sites, but the function was not known. There are three SNP sites located both in the upstream region of AFUA_6G08500 and in the downstream region of AFUA_6G08510. AFUA_6G08500 encodes a phosphoglycerate mutase family protein, and AFUA_6G08510 encodes cell wall glucanase. Details of these 61 SNP sites are given in the Appendix (Supplementary Table S3).

### Enrichment analysis

**FIGURE 7:**
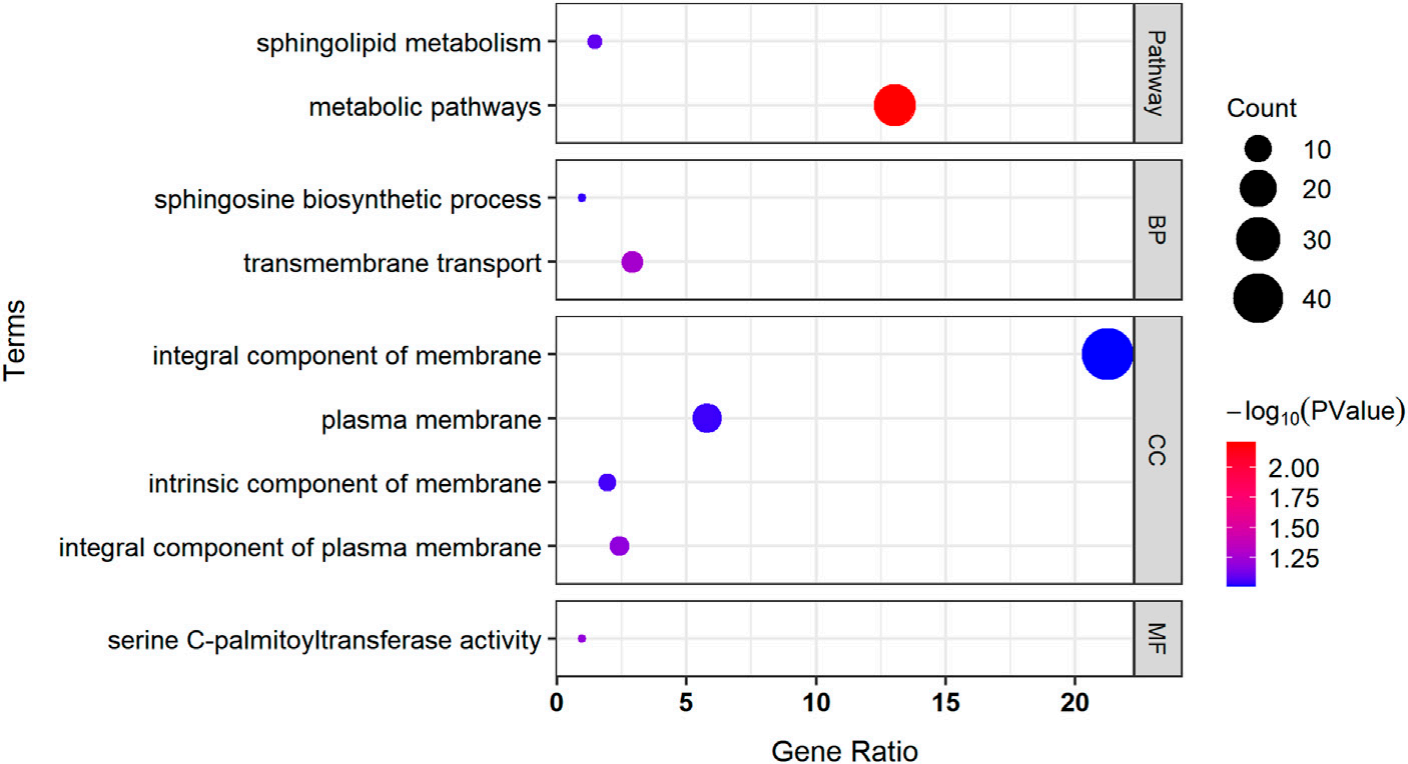
Pathway enrichment analysis and GO enrichment analysis of associated genes. BP, biological process; CC, cellular component; MF, molecular function.

We used the DAVID website to perform GO and KEGG pathway enrichment analyses on 216 identified genes (Figure 7). Two highly significant pathways were obtained with 27 genes in the metabolic pathways and three genes in the sphingolipid metabolism pathway. GO enrichment analysis identified two more significant biological processes, with six genes involved in transmembrane transport and two genes involved in the sphingosine biosynthetic process. The four significant cellular components were all associated with the cell membrane, with the products of 44 genes acting on the integral component of the membrane, the products of five genes acting on the integral component of the plasma membrane, the products of four genes acting on the intrinsic components of the membrane, and the products of 12 genes acting on the plasma membrane. The most significant molecular function was serine C-palmitoyltransferase activity.

## Discussion

In order to expand the type of genetic variants detected in the GWAS, the present study used *k*-mers as genetic markers that can tag a broad range of polymorphisms independent of a reference genome and then directly linked *k*-mers associated with phenotypes to specific genomic regions. Using this approach, we reanalyzed the AMB resistance in 98 *A. fumigatus* strains. Associations identified with *k*-mers not only recapitulate those found with SNPs but also discover new associations with indel.

Different from the majority of published *k*-mer-based GWAS that directly used the present/absent state of *k*-mers in read sequences, our approach was based on the *k*-mer copy numbers in the genome, which avoids the influence of sequencing depth. Due to the lack of alignment, the types and genomic positions of *k*-mers are initially unknown. Thus, the *k*-mers’ length is set by the need to maintain *k*-mer homology. Longer *k*-mers preserve *k*-mer homology but increases the proportion carrying multiple variants. Thus, we first choose a minimum length that can maintain *k*-mer homology. This value is determined by genome size. For *A. fumigatus*, the optimal *k*-mer length was 19 bases. At *k* = 19, 97.8% *k*-mers of the total were unique. The remaining *k*-mers might derive from duplications and the possible presence of multiple copies of mobile elements within the genome which would not be unique even with longer *k*-mers. To avoid the effect of repetitive sequences, only unique *k-*mers were retained, and all of the remaining *k*-mers possess two allelic contrasts: presence or absence.

Association analysis showed that 688 variants were linked with AMB resistance, and most of them were SNP sites. Among them, 12 SNP sites were also highly associated with AMB resistance in the SNP-based GWAS. A non-synonymous mutated SNP is located on AFUA_2G02360, the encoding gene of the putative UDP-glucose: glycoprotein glucosyltransferase (UGGT), which is central to the process of facilitating glycoprotein folding and quality control (Dejgaard et al., 2004). Deletion of this gene causes alterations in the cell walls of many fungi, resulting in resistance to antifungal drugs (Meaden et al., 1990; Herrero et al., 2004). The *k*-mer-based GWAS also found some indel, showing a stronger association with AMB resistance than SNP sites, and a significant associated indel is present in the exonic region of AFUA_7G05160, the product of which is the fumarylacetoacetate hydrolase (FAH) family protein. FAH deficiency leads to the upregulation of some ROS scavenging genes in *Arabidopsis* (Huang et al., 2018). It has been reported that the oxidation–reduction process was relevant to AMB resistance in fungi (Jukic et al., 2017). There are both significant SNP and indel mutations in the upstream region of AFUA_6G03110, encoding a putative C6 transcription factor. Some members of this transcription factor family affected ergosterol synthesis and further affect azole resistance in *A. fumigatus* (Paul et al., 2019).

The KEGG pathway enrichment analysis identified two pathways with high significance: metabolic pathways and sphingolipid metabolism. The most significant molecular function in the GO enrichment analysis was serine C-palmitoyltransferase activity, the first step in sphingolipid biosynthesis catalyzed by serine palmitoyltransferase (Miyake et al., 1995). A recent study on *Saccharomyces cerevisiae* and *Candida albicans* has shown that sphingolipids can modulate fungal resistance to AMB and that the inhibition of sphingolipid biosynthesis sensitizes cells to AMB (Sharma et al., 2014). GO enrichment analysis identified all four significant cellular components were associated with the cell membrane. Biofilm formation contributes to multiple drug resistance (MDR), and in recent years, several studies have shown that biofilms play an important role in the mechanism of fungal MDR (Bojsen et al., 2014; Lagree et al., 2018). The ability of *C. albicans* to form biofilms is an important contributor for antifungal treatment resistance (Mba and Nweze, 2020; Mourer et al., 2021). In addition, four protein transporter family genes were also found, namely, two ABC protein family genes: AFUA_3G09680 (ABC efflux transporter, putative) and AFUA_5G00790 (ABC multidrug transporter, putative), and two MFS protein transporter family genes: AFUA_1G17160 (MFS transporter, putative) and AFUA_2G04070 (MFS transporter, putative). Overexpression of intracellular ATP-binding cassette (ABC) and MDR efflux transporter protein genes, which mainly promote the superfamily (MFS), is an important cause of multidrug resistance in fungi (Gulshan and Moye-Rowley, 2007). Overexpression of the drug efflux pump also led to azole resistance in *A. fumigatus* (Nascimento et al., 2003; Meneau et al., 2016).

In conclusion, we propose a *k*-mer-based GWAS to identify not only SNPs but also indels and structural variants associated with AMB resistance in *A. fumigatus*. Compared to SNPs, indels showed a stronger association with AMB resistance. The new associated variants identified in this study will provide a basis for future research on the mechanism of AMB resistance in *A. fumigatus* and contribute to the development of relevant molecular markers for clinical diagnosis.

## Data Availability

The datasets presented in this study can be found in online repositories. The names of the repository/repositories and accession number(s) can be found in the article/Supplementary Material.
